# Otitis media in coronavirus disease 2019: a case series

**DOI:** 10.1017/S0022215120002741

**Published:** 2021-01-07

**Authors:** N Raad, J Ghorbani, N Mikaniki, S Haseli, M Karimi-Galougahi

**Affiliations:** 1 Department of Otolaryngology, Masih Daneshvari Hospital, Tehran, Iran; 2 Chronic Respiratory Disease Research Center, National Research Institute of Tuberculosis and Lung Diseases, Shahid Beheshti University of Medical Sciences, Tehran, Iran; 3 Department of Otolaryngology, Iranshahr University of Medical Sciences, Sistan and Baluchestan, Iran

**Keywords:** COVID-19, Coronavirus, Otitis Media, Otalgia, Hearing Loss

## Abstract

**Background:**

Manifestations of the coronavirus disease 2019 in ENT include sore throat, rhinorrhoea, anosmia and dysgeusia. Whether coronavirus disease 2019 causes otitis media is not known.

**Objective:**

To assess the presence of otitis media in a series of patients with confirmed coronavirus disease 2019 and ENT symptoms.

**Methods:**

The study included patients with coronavirus disease 2019, confirmed on polymerase chain reaction assay, who had otological (e.g. otalgia, otorrhoea, hearing loss) or other ENT (e.g. anosmia, dysgeusia) manifestations of coronavirus disease 2019, in two tertiary referral hospitals in Iran. Patients were excluded if they had a background of otological problems including previous acute otitis media, chronic otitis media, otological surgery, and trauma or radiotherapy to the head and neck.

**Results:**

Otitis media was found in eight patients with coronavirus disease 2019 and no background of otological problems. Six patients had middle-ear effusion, three had typical signs of acute otitis media, and one had a tympanic membrane perforation. Most patients had hearing loss; conductive hearing loss and mild sensorineural hearing loss at high frequencies were the underlying mechanisms.

**Conclusion:**

Otitis media should be considered a manifestation or associated symptom of coronavirus disease 2019 during the current pandemic.

## Introduction

Coronavirus disease 2019 (Covid-19) frequently manifests with a spectrum of symptoms, most commonly fever, cough, myalgia, fatigue and shortness of breath.^1^ ENT symptoms, including sore throat, rhinorrhoea, anosmia, dysgeusia, vertigo and hearing loss, have also been reported.^1,2^ The current study presents a series of patients, with no background history of otological problems, who presented with otitis media and conductive hearing loss in the context of confirmed Covid-19.

## Case studies

### Patient one

A 38-year-old man with ‘the worst cold of his life for the last 2 weeks’ presented with a chronic non-productive cough and mild dyspnoea on exertion. Additionally, he complained of a 4-day history of hearing loss, and a sense of fullness and pressure in his ears. There were bilateral coarse crackles and wheeze on lung auscultation. On otoscopy, tympanic membrane bulging and purulent effusion were observed. Bilateral consolidations with bronchiolectasis in the peripheral locations were identified in an organising pneumonia pattern on non-contrast spiral computed tomography (CT). Polymerase chain reaction assay of an oropharyngeal swab was positive for Covid-19. The patient was treated as an out-patient and all the symptoms except for anosmia resolved within the following two weeks. The olfactory sense began to return after three months.

### Patient two

A 35-year-old woman presented with sudden-onset anosmia, which had commenced 7 days previously. She did not have fever, systemic symptoms, cough, dyspnoea or otological symptoms. There were coarse crackles on auscultation of the left upper lung lobe. The head and neck examination revealed no signs of nasal congestion or discharge; however, bilateral middle-ear effusion was observed on otoscopy. A chest CT scan revealed consolidation in the lingula. Coronavirus disease 2019 was confirmed on the polymerase chain reaction assay of a nasopharyngeal swab. The patient did not develop otological symptoms during follow up. Anosmia persisted for a few weeks before gradually improving.

### Patient three

**Fig. 1.**
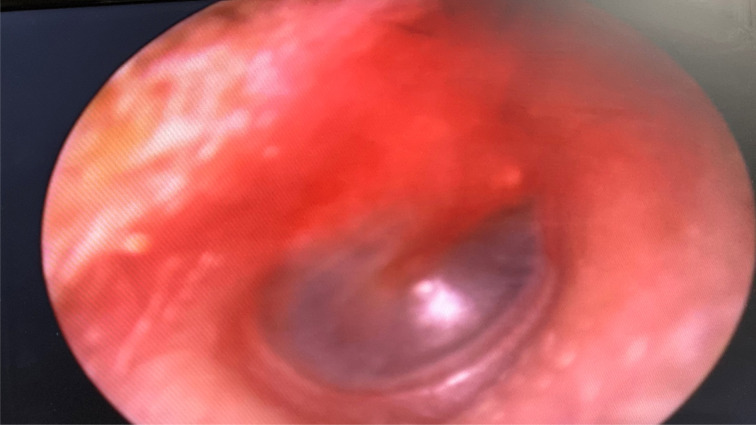
Erythema of the tympanic membrane in a 35-year-old woman with coronavirus disease 2019.

A 35-year-old woman was admitted with a 2-week history of cough and progressive dyspnoea. A few days into her admission, the patient developed unilateral earache and hearing loss. The otoscopic examination revealed a distinctly red tympanic membrane (Figure 1). There were bilateral pulmonary changes characteristic of Covid-19 on a CT scan of the chest, with Covid-19 confirmed by positive polymerase chain reaction assay of a nasopharyngeal swab. The patient's otalgia and hearing loss gradually improved over a few days. She was discharged from the hospital two weeks after admission.

### Patient four

**Fig. 2.**
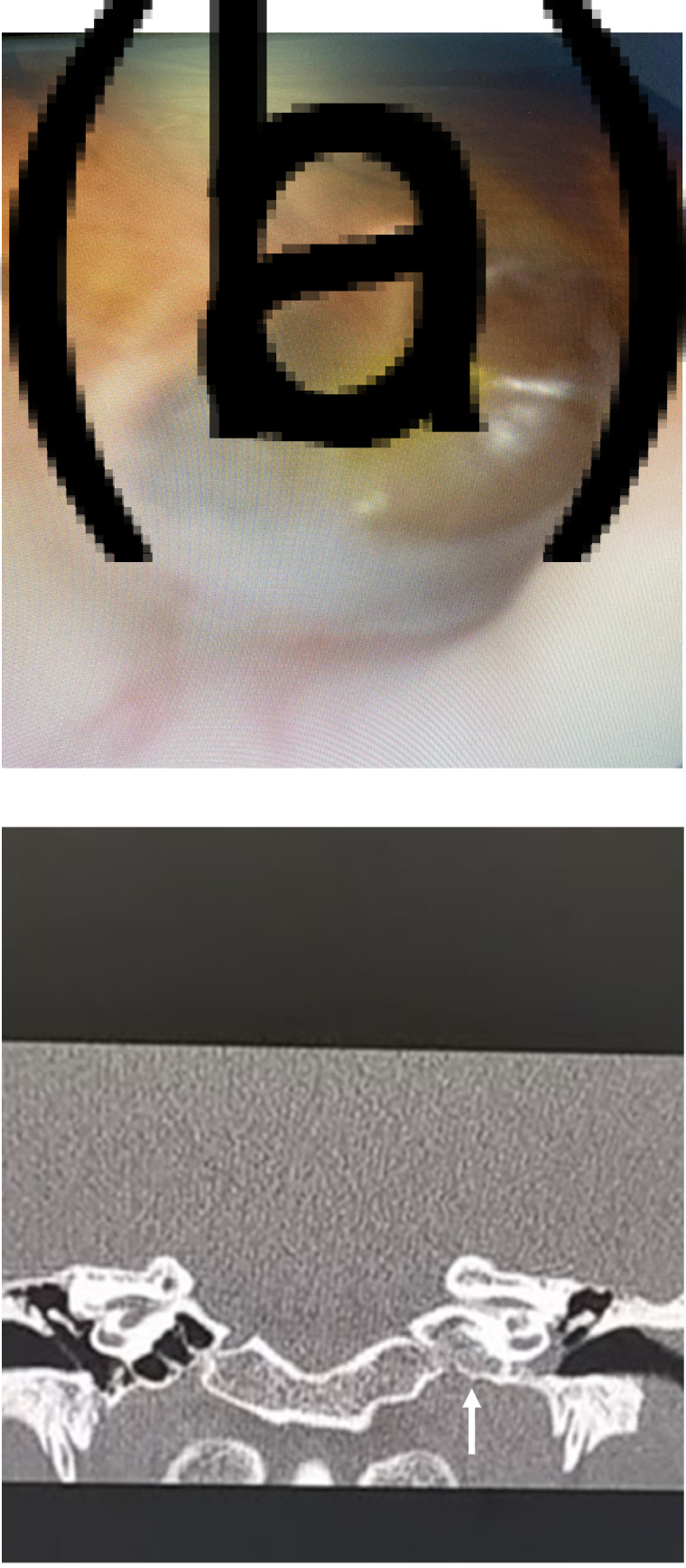
(a) Effusion in the left middle ear with an air–fluid level and tympanic membrane bulging on otoscopy. (b) Opacification of the left middle air cavity (arrow) on coronal high-resolution computed tomography of the temporal bones. Polymerase chain reaction assay of the middle-ear fluid was positive for coronavirus disease 2019.

A 20-year-old woman presented with left-sided ear pain and hearing loss. She had recently been in close contact with a family member with confirmed Covid-19, but reported no systematic or respiratory symptoms. Ear examination revealed effusion in the left middle ear, an air–fluid level and severe bulging of the tympanic membrane (Figure 2a). The physical examination findings were otherwise normal. On coronal, high-resolution CT images of the temporal bones, opacification of the left middle air cavity was observed (Figure 2b). Computed tomography of the chest was normal. Myringotomy was performed in light of the tympanic membrane bulging, to relieve pressure and earache. Polymerase chain reaction testing of an oropharyngeal swab was negative for Covid-19; however, the polymerase chain reaction testing performed on the middle-ear fluid was positive for Covid-19. The patient did not develop symptoms of Covid-19 during follow up. The hearing in the left ear returned to normal within a few weeks.

### Patient five

**Fig. 3.**
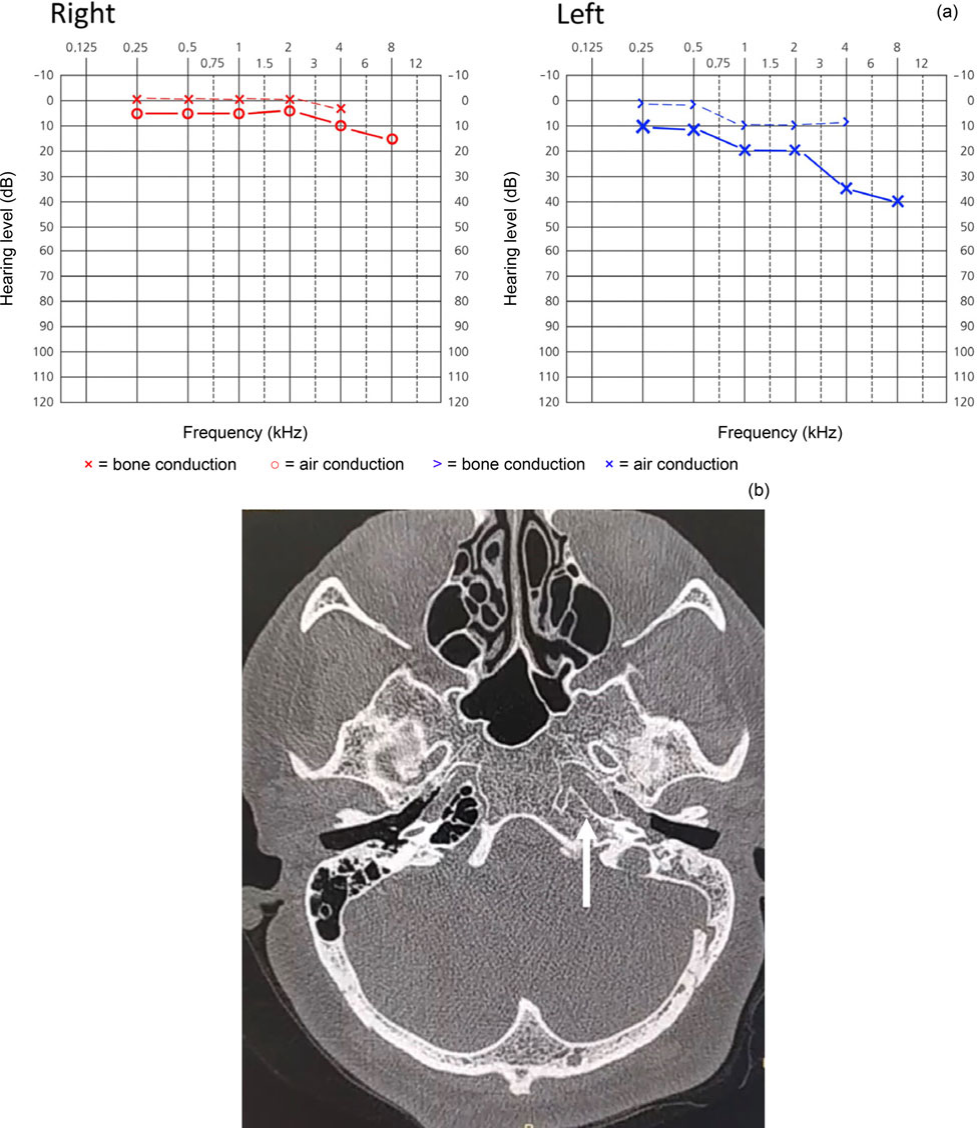
(a) An audiogram revealed left-sided conductive hearing loss and mild sensorineural loss at high frequencies. (b) Axial high-resolution computed tomography of the temporal bones revealed opacification of the left middle air cavity (arrow), indicating otitis media.

A 22-year-old woman with a 7-day history of a non-productive cough presented with left-sided ear pain, aural fullness, hearing loss and a sensation of ‘ear popping’ for the previous 24 hours. There were crackles and asymmetric vocal resonance on chest auscultation on the right side. Otoscopic examination revealed decreased mobility of the left tympanic membrane, with a bulging contour, hypervascularity and purulent middle-ear effusion. The audiogram revealed conductive hearing loss (15 dB) on the left side, with mild sensorineural hearing loss at high frequencies (Figure 3a). Axial high-resolution CT images of the temporal bones showed opacification of the left middle air cavity, suggestive of otitis media (Figure 3b). On high-resolution CT of the lungs, there were right-sided, multifocal, ill-defined ground-glass opacities characteristic of Covid-19. Polymerase chain reaction testing of an oropharyngeal swab was negative for Covid-19, but the result was positive for a nasopharyngeal swab. With out-patient treatment, all the patient's symptoms subsided within a few weeks.

### Patient six

A 25-year-old woman with a non-productive cough for the previous 3 weeks presented with right-sided hearing loss and otalgia that had started a week earlier. There were mixed coarse and fine crackles on auscultation of the right lung. Otoscopic examination revealed serous otitis media with decreased tympanic membrane movement. On high-resolution CT of the lungs, there were right-sided foci of ground-glass opacity, consistent with viral pneumonia. Polymerase chain reaction assays performed on the oropharyngeal and nasopharyngeal swabs were positive for Covid-19. She received out-patient treatment. There were major improvements in the cough and otological symptoms within two weeks, and all symptoms had subsided within a month.

### Patient seven

A 22-year-old woman presented with sudden loss of smell and taste, and left-sided otalgia and hearing loss, experienced for a week. She did not have any other symptoms (i.e. no fever, cough or dyspnoea). Otoscopic examination revealed typical signs of otitis media with effusion (OME) and an air–fluid level. She had a unilateral C type tympanogram. Axial non-contrast CT of the lungs revealed patchy foci of ground-glass opacities in the right upper lobe. A polymerase chain reaction assay performed on the oropharyngeal swab was positive for Covid-19. She was managed as an out-patient. Her otological symptoms started to improve after 2 days. Otoscopic examination and tympanogram findings became normal after 30 days.

### Patient eight

**Fig. 4.**
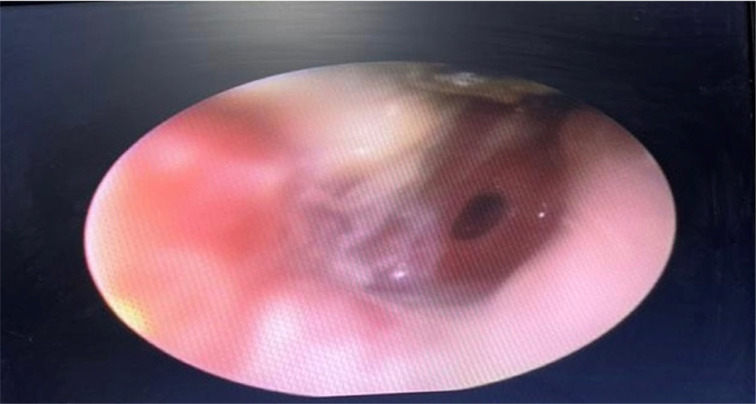
Acute otitis media with a central tympanic membrane perforation in a 45-year-old woman with coronavirus disease 2019.

A 45-year-old woman presented with a 4-day history of severe acute otalgia, ear fullness and hearing loss. She had experienced a mild cough but no dyspnoea during the previous week. There were bilateral coarse crackles on auscultation of the lungs. Ear examination revealed central tympanic membrane perforation with purulent otorrhea (Figure 4). Axial, non-contrast CT scans of the chest revealed bilateral, patchy ground-glass opacities in the periphery of the lower lobes. A polymerase chain reaction assay performed on the nasopharyngeal swab was positive for Covid-19. She was treated as an out-patient. All her symptoms resolved within a week. The perforation was observed to have healed on otoscopic examination conducted at a six-week follow-up visit.

## Discussion

In this case series, we report otitis media as a manifestation or associated symptom of Covid-19.

Otitis media, or middle-ear inflammation, is categorised as: acute otitis media, OME or chronic suppurative otitis media.^3^ The present case series includes eight patients who presented over a two-month period during the Covid-19 pandemic. Six of the eight patients had otalgia, and seven patients had hearing loss. Middle-ear effusion was evident on otoscopic examination in six patients. Three patients had typical signs of acute otitis media; one patient had acute otitis media with a tympanic membrane perforation. Two patients had olfactory dysfunction. Coronavirus disease 2019 was confirmed by typical changes on CT of the chest, and/or positive polymerase chain reaction assays on nasopharyngeal or oropharyngeal swabs. Interestingly, in one patient, polymerase chain reaction testing was positive for the middle-ear effusion and negative for the oropharyngeal swab.

We suggest that the concomitant occurrence of otitis media in these patients could be either a manifestation or complication of Covid-19. Acute otitis media and OME follow a seasonal pattern, with the incidence being highest during autumn and winter, and lowest during spring and summer, in parallel to the incidence of upper respiratory infections.^3^ The patients in this case series presented with otitis media in the second and third month of spring, when a low incidence of otitis media is expected. In addition, acute otitis media and OME are most common in young children,^3^ while all the patients in this case series were adults, without any background history of otolaryngological issues or nasopharyngeal pathology.

Viruses, including respiratory syncytial virus, rhinovirus, adenovirus, coronavirus, bocavirus, influenza virus, parainfluenza virus, enterovirus and human metapneumovirus, are known causes of upper respiratory infections and can induce acute otitis media.^3^ Viruses can be the sole infective cause of acute otitis media, or play a role in co-infection with bacteria and, rarely, with other viruses.^4^ Viral infections can disrupt immune function, and reduce the normal mucociliary clearance of mucosal cells by changing the properties of the nasopharyngeal mucus and the Eustachian tube, leading to negative middle-ear pressure.^3^ Negative middle-ear pressure in turn predisposes the middle ear to effusion formation, and secondary bacterial or viral infection.^4^

• This study reports otitis media in eight patients with confirmed coronavirus disease 2019 (Covid-19) and no background of otological problems
• Middle-ear effusion was detected in six patients
• Hearing loss occurred in most patients; conductive loss and mild sensorineural loss at high frequencies were the underlying mechanisms
• Otitis media should be considered a possible manifestation and/or associated symptom of Covid-19 during the current pandemic

The angiotensin-converting enzyme-2 (ACE2) receptor is the cellular entry route for severe acute respiratory syndrome coronavirus-2 (SARS-CoV-2). High levels of ACE2 are expressed in nasal respiratory epithelium goblet, basal and ciliated cells.^5^ Because of the enriched populations of ciliated cells, glands and goblet cells in the inferior part of the Eustachian tube,^6^ we suggest that this portion of the tube can be a potential route for SARS-CoV-2, causing infection of the middle ear.

## Conclusion

To the best of our knowledge, only a single case of otitis media in the setting of Covid-19 has thus far been reported.^7^ The current study presents the largest series of patients with otitis media and Covid-19. Interestingly, otitis media was the first manifestation of Covid-19 in some patients in this case series. Thus, we recommend that during the current pandemic, the presence of otitis media should alert clinicians to the possibility of Covid-19.
